# Metabolic risk factors of cognitive impairment in young women with major psychiatric disorder

**DOI:** 10.3389/fpsyt.2022.880031

**Published:** 2022-07-29

**Authors:** Chuanjun Zhuo, Wei Liu, Ronghuan Jiang, Ranli Li, Haiping Yu, Guangdong Chen, Jianmin Shan, Jingjing Zhu, Ziyao Cai, Chongguang Lin, Langlang Cheng, Yong Xu, Sha Liu, Qinghua Luo, Shili Jin, Chuanxin Liu, Jiayue Chen, Lina Wang, Lei Yang, Qiuyu Zhang, Qianchen Li, Hongjun Tian, Xueqin Song, China multiple organs damage in the mental disorder (CMODMD) Group

**Affiliations:** ^1^Department of Psychiatry, Tianjin Fourth Center Hospital, Tianjin, China; ^2^Department of Psychiatry, First Affiliated Hospital of Zhengzhou University, Zhengzhou, China; ^3^Henan Psychiatric Transformational Research Key Laboratory, Zhengzhou University, Zhengzhou, China; ^4^Multiple Organs Damage in the Mental Disorder (MODMD) Center of Wenzhou Seventh Hospital, Wenzhou, China; ^5^Department of Psychiatry, Tianjin Anding Hospital, Tianjin, China; ^6^Department of Psychiatry, The First Affiliated Hospital of Harbin Medical University, Harbin, China; ^7^Department of Psychiatry, General Hospital of PLA, Beijing, China; ^8^Key Laboratory of Psychiatric-Neuroimaging-Genetic and Cor-morbidity, Tianjin Mental Health Center of Tianjin Medical University, Tianjin Anding Hospital, Tianjin, China; ^9^Inpatient Department of Wenzhou Seventh Peoples Hospital, Wenzhou, China; ^10^Department of Psychiatry, First Hospital/First Clinical Medical College of Shanxi Medical University, Taiyuan, China; ^11^Department of Psychiatry, The First Affiliated Hospital of Chongqing Medical University, Chongqing, China; ^12^Inpatient Department, Shandong Daizhuang Hospital, Jining, China; ^13^Department of Psychiatry, Tianjin Fourth Center Hospital, Tianjin, China; ^14^Department of Psychiatry, Tianjin Anding Hospital, Tianjin, China; ^15^Department of Psychiatry, Yanan Fifth Hospital, Yan'An, China; ^16^Department of Psychiatry, Tianjin Anning Hospital, Tianjin, China; ^17^Department of Psychiatry, Hebei Fifth Peoples Hospital, Shijiazhuang, China; ^18^Key Laboratory of Multiple Organ Damage in Patients With Mental Disorder, Tianjin Fourth Center Hospital of Tianjin Medical University, Nankai University Affiliated Tianjin Fourth Center Hospital, Tianjin, China; ^19^Department of Psychiatry, The First Affiliated Hospital of Zhengzhou University, Zhengzhou, China; ^20^Henan Psychiatric Transformational Research Key Laboratory, Zhengzhou University, Zhengzhou, China

**Keywords:** major psychiatric disorder, metabolic syndrome, HbA1c, cognitive impairment, risk factors

## Abstract

**Background:**

Cognitive performance improves clinical outcomes of patients with major psychiatric disorder (MPD), but is impaired by hyperglycemia. Psychotropic agents often induce metabolism syndrome (MetS). The identification of modifiable metabolic risk factors of cognitive impairment may enable targeted improvements of patient care.

**Objective:**

To investigate the relationship between MetS and cognitive impairment in young women with MPD, and to explore risk factors.

**Methods:**

We retrospectively studied women of 18–34 years of age receiving psychotropic medications for first-onset schizophrenia (SCH), bipolar disorder (BP), or major depressive disorder (MDD). Data were obtained at four time points: presentation but before psychotropic medication; 4–8 and 8–12 weeks of psychotropic therapy; and enrollment. MATRICS Consensus Cognitive Battery, (MCCB)—based Global Deficit Scores were used to assess cognitive impairment. Multiple logistic analysis was used to calculate risk factors. Multivariate models were used to investigate factors associated with cognitive impairment.

**Results:**

We evaluated 2,864 participants. Cognitive impairment was observed in 61.94% of study participants, and was most prevalent among patients with BP (69.38%). HbA1c within the 8–12 week-treatment interval was the most significant risk factor and highest in BP. Factors in SCH included pre-treatment waist circumference and elevated triglycerides during the 8–12 weeks treatment interval. Cumulative dosages of antipsychotics, antidepressants, and valproate were associated with cognitive impairment in all MPD subgroups, although lithium demonstrated a protect effect (all *P* < 0.001).

**Conclusions:**

Cognitive impairment was associated with elevated HbA1c and cumulative medication dosages. Pre-treatment waist circumference and triglyceride level at 8–12 weeks were risk factors in SCH. Monitoring these indices may inform treatment revisions to improve clinical outcomes.

## Introduction

Schizophrenia (SCH), bipolar disorder (BP) and major depressive disorder (MDD) are categorized as major psychiatric disorders (MPD) (1). SCH, BP, and MDD are prevalent among young women (18–34 years of age) (2–4). Both MPD and complications of therapy such as drug-induced metabolic syndrome (MetS) and anticholinergic side effects impede the total functioning of these young women (3–8). Sequelae include impaired cognition, reproductive function, and community engagement. Cognitive impairment poses a key barrier to recovery by reducing treatment compliance; disrupting life style; degrading community functioning; and worsening regression; thus, leading to a vicious cycle that furthers psychomorbidity and social dysfunction, ending in lifelong disability (1–10). Against this background, interest in the mitigation of cognitive impairment has grown substantially. Since the 1990s, cognitive impairment in patients with MPD has become a focus of clinical practice. An increasing number of psychiatrists believe that improvement of cognition is a pivotal therapeutic target to enhance community functioning (11–15). Although cognitive impairment may precede (based on the neurodevelopment hypothesis) or complicate MPD (based on the neurodegeneration hypothesis), mitigation of cognitive impairment should be given a high priority (16–18). Hence, investigation of the risk factors of cognitive impairment and the search for effective therapies have become hot spots of psychiatric research.

MetS is defined in women by presence of 3 of 5 criteria: (1) waist circumference ≥88 cm, (2) fasting blood glucose ≥100 mg/dl, (3) systolic blood pressure ≥130 mm Hg or diastolic blood pressure ≥85 mm Hg, (4) serum triglyceride level ≥150 mg/dl, and (5) serum HDL cholesterol level <50 mg/dl (19). Mets has complicated the therapies of SCH, BP, and MDD (20–22). Drugs that may induce MetS include second-generation antipsychotic agents (23), valproate (24), and several mood stabilizers (although the definition of mood stabilizer is controversial) (25). These agents, especially second-generation antipsychotics (26) and selective serotonin reuptake inhibitor antidepressants induce hyperglycemia before the onset of MetS (27).

During the past 20 years, multiple studies of diabetes and pre-diabetes have associated hyperglycemia with cognitive impairment. For example, our previous study demonstrated that clozapine induced pre-diabetes/diabetes in 75.57% of recipients despite the addition of metformin to their treatment regimens (28, 29). Furthermore, clozapine-induced pre-diabetes/diabetes was associated with both reduced treatment benefit and cognitive impairment (30). Similar observations have been made in multiple studies focused on altered cognition in hyperglycemic patients, particularly those with prediabetes/diabetes (31). Cognitive impairments may be explained by hyperglycemia-induced cerebral oxidative stress, associated neuroendocrine disturbances, and cerebral structural and functional disturbances (32–38). Other indirect mechanisms may also play key roles in the relationship between psychotropic medications, hyperglycemia, and cognitive impairment. However, to the best of our knowledge, few studies have reported risk factors of cognitive impairment (39–49). What must be emphasized is that HbA1c, which accounts for 70% of glycosylated hemoglobin, can accurately reflect glycemic control over the preceding 8–12 weeks. However, HbA1c, as a stable index, cannot confirm the diagnosis of MetS (50–53). Hence, in the future, a well-designed study should be conducted to explore the value of HbA1c in the diagnosis of MetS.

The above-mentioned studies suggest that the relationship between psychotropic agents-hyperglycemia-cognitive impairment may be caused directly by the mechanisms of therapeutic agents, although this hypothesis requires further study for clarification. Nonetheless, from the clinical perspective, multiple studies associate psychotropic-induced hyperglycemia with cognitive impairment.

Drug-induced hyperglycemia is an obvious risk factor for poor clinical outcomes; consequently, the identification of a biomarker of psychotropic-induced hyperglycemia may facilitate early targeted intervention to improve clinical outcomes of patients with MPD. Our previous study demonstrated the difficulty of conducting a prospective study to investigate the relationship between glycemic control and treatment effect; consequently, we conducted a retrospective study to investigate the relationship between psychotropic medication exposures, indices of hyperglycemia, and cognitive impairment in young women with MPD. We hypothesized that (1) hyperglycemia is associated with cognitive impairments in young women with MDP; (2) SCH, BP, and MDD may exhibit different ORs for cognitive impairment due to disease-specific psychomorbidities; (3) some therapeutic agents may preserve cognitive function, consistent with previous studies that confirmed neuroprotective effects. Although the strengths of evidence of retrospective studies are often inferior to those of prospective trials, our findings at least provide several pivotal topics for further investigation.

## Materials and methods

### Study design and participants

In this retrospective cohort study, participants were recruited by senior psychiatrists at the outpatient departments of 10 psychiatric hospitals located in the north, south, east, and west regions of China. Recruitment was conducted over 2 months (1st July to 31st August 2021). Inclusion criteria were (1) female sex; (2) age range of young adulthood (18–34 years old); (3) diagnosis of SCH, BP, or MDD according to DSM-IV-TR criteria (54); (4) the first onset of either SCH, BP, or MDD of at least 18 months duration, (5) full insight into their mental illness and treatment, with insight confirmed by the Birchwood Insight Scale (BIS) (55) and Beck Cognitive Insight Scale (BCIS) (56), (6) recollection of their clinical trajectory over the preceding 18 months and normal memory ability assessed by the Chinese version of the Wechsler Memory Scale-third version (57), (7) medical records that documented FBS, PBG-2h, HbA1c, triglyceride and HDL cholesterol levels; blood pressure; and waist circumference at four time points: at the time of presentation but before the initiation of psychotropic medication; at 4–8 weeks of psychotropic therapy; at 8–12 weeks of psychotropic therapy; and study enrollment. (8) documentation of medication dosages administrated in the 18 months preceding enrollment to enable calculation of cumulative dose; (9) MATRICS Consensus Cognitive Battery (MCCB) scores (including 7-dimension scores) at the four time points (58); (10) volunteered to participate in this study and provide socio-demographic data. Exclusion criteria were: (1) did not volunteer to participate in this study, (2) could not clearly remember the clinical trajectory of their illness over the preceding 18 months, (3) history of pregnancy or abortion during the preceding 18 months, (4) histories of neurological disease, other organ system disorders, or substance abuse in the preceding 18 months; (5) other psychiatric diagnoses (e.g., personality disorders), (6) life events that can provoke stress reactions during the preceding 18 months, (7) inability of their female guardian to provide reliable information to assist the patients in providing details of their illness, menstrual status, and other needed data, (8) history of alcohol or nicotine use.

We acquired demographic and clinical data including the categories of mental disorders, cumulative drug dosage, cognitive performance at the time of psychiatric diagnosis. Blood pressure; waist circumference; and levels of fasting blood sugar (FBS), 2-h postprandial blood glucose (PBG-2h), HbA1c, and HDL cholesterol recorded at four time points were obtained from medical insurance records. Ethics approval was granted from the Ethics Committee of Tianjin Fourth Center Hospital of Tianjin Medical University (approval number: ZC-R-0001).

### Procedures

#### Tools

Official medical records were reviewed to exclude histories of neurologic and other organ system diseases, substance abuse, and pregnancy or abortion in the 18 months preceding enrollment and to confirm therapeutic agent dosage. DSM-IV (54) and SCID-I/P (59) were adopted to define the psychiatric diagnosis. Mental illnesses were defined by core symptoms from the first episode to the time of enrollment. The BIS (55) and BCIS (56) were adopted to assess insight. The Chinese version of the Wechsler Memory Scale-Fourth edition was used to assure normal memory ability (57). Chlorpromazine (60) and fluoxetine equivalents (61) were used to record the cumulative dosage of antipsychotic or antidepressant agents over the preceding 18 months, respectively. Sodium valproate equivalent was used to record the cumulative dosage of the mood stabilizers during the preceding 18 months. Diazepam equivalent was used to calculate the cumulative dosage of the anxiolytics and sleeping agents over the preceding 18 months (62). Body Mass Index (BMI) (63) was adopted to assess obesity. The double antibody radioimmune method was used to assess prolactin, estradiol, progesterone, and testosterone levels. A Roche Cobas 6000 analyzer (Roche Diagnostics GmbH, Mannheim, Germany) was used to assess FBS and PBG-2h (64). A Hitachi 7600 automated analyzer (H-7600, Hitachi High-Technologies, Tokyo, Japan) was adopted to assess HbA1c (65) and blood cholesterol and triglyceride levels (66). Pregnancy testing was used to rule out pregnancy at the time of enrollment (67).

### Outcome definition

We compared the seven dimensions scores of the MCCB obtained before the administration of therapeutic agents and at enrollment. According the MCCB rules, GDS scores were used to define cognitive impairment (68). Mild impairment was defined by GDS ≥1, moderate impairment by GDS ≥2; and severe impairment by GDS ≥3.

### Statistical analysis

Statistical analyses were made using SAS statistical software (version 9.3, SAS Institute, Cary, NC, USA). Data were expressed as mean ± standard deviation (normally distributed data) or median ± interquartile range (non-normal data) for continuous variables, and as numbers and percentages for categorical variables. Associations of clinical-demographic characteristics with MPD incidence were evaluated using univariate and multivariate logistic regression models and expressed with odds ratios (ORs) and 95% confidence intervals (CI) in the overall population and by psychiatric disease-specific analyses. Multivariate logistic models were built first by adjusting for factors found to be significant in univariate analysis (*P* < 0.02), and then limiting the final multivariate models to risk factors or confounders that were statistically significant.

### Role of the funding source

The funders of this study had no role in study design, data collection, data analysis, data interpretation, or writing of the report. Chuanjun Zhuo and Xueqin Song had full access to all the data and had final responsibility for the decision to submit for publication. All the data and evidence obtained in this study (picture and voice recording of data collection) can be provided by Xueqin Song, Ronghuan Jiang, Ranli Li, Haiping Yu, Guangdong Chen, Jianmin Shan, Jingjing Zhu, Ziyao Cai, Chongguang Lin, Langlang Cheng, Guangdong Chen, Yong Xu, Sha Liu, Qinghua Luo, Shili Jin, Chuanxin Liu, Qiuyu Zhang, Lei Yang, Jiayue Chen, Qianchen Li, Lina Wang, and Hongjun Tian. Although Shandong Qilu Pharmaceutical Co., Ltd; Jiangsu Haosen Pharmaceutical Co., Ltd; Jiangsu Enhua Pharmaceutical Co., Ltd; Beijing Yimin Pharmaceutical Co., Ltd, Sinopharm holding medical technology (Tianjin) Co., Ltd and Beijing Jingdong Century Trading Co., Ltd sponsored this study, we used the uniform equivalent method to calculate cumulative therapeutic dosages, thereby obviating information bias.

## Results

We recruited 3,500 study candidates, of whom 2,864 (81.83%, 2,864/3,500) met the inclusion criteria as confirmed by primary medical records provided by their medical insurance institutions. Our study cohort was comprised of 787 patients with BP, 899 with MDD, and 1,178 with SCH. Age, education level, and illness duration were similar among these three groups.

Cognitive impairment was observed in 61.94% (1,774/2,864). Subgroup analysis disclosed that the prevalence of cognitive impairment was highest in patients with BP at 69.38% (546/787); followed by SCH at 62.73% (739/1,178) and MDD at 54.39% (489/899). Univariate analysis demonstrated that age and cumulative dose of aripiprazole were significantly associated with cognitive impairment, but were not associated after multivariate analysis. Multivariate analysis demonstrated that HbA1c level within the 8–12-week treatment interval was the most significant risk factor for cognitive impairment, especially for visual learning ability, in the entire cohort. The odds ratio (OR) of HbA1c in the total cohort was 8.45 [95% confidence interval (95% CI): 6.30–9.87; *P* < 0.00010] and was highest in patients with BP (OR 9.95, 95% CI: 7.40–12.46; *P* < 0.0001); followed by patients with SCH (OR 8.88; 95% CI: 5.29–14.97; *P* < 0.0001) and MDD (OR 8.29; 95%CI: 4.88–12.33; *P* < 0.0001). Among patients with BP, ORs for FBS and PBG-2h within the treatment duration were 6.52 (95% CI: 4.85–8.91; *P* < 0.0001) and 7.27 (95% CI: 5.68–9.89; *P* < 0.0001), respectively. Patients with MDD exhibited ORs of FBS and PBG-2h of 4.99 (95% CI: 2.25–9.60; *P* < 0.0001) and 3.68 (95% CI: 1.53–7.49; *P* < 0.0001), respectively. The ORs of FBS and PBG-2h in patients with SCH were 6.59 (95% CI: 4.28–9.99; *P* < 0.0001), and 8.20 (95% CI: 6.40–10.00; *P* < 0.0001), respectively. A more notable finding was that the OR of triglyceride within the 8–12 weeks treatment interval in patients with SCH was significant (OR 6.96; 95% CI: 4.19–9.97; *P* < 0.0001). Waist circumference before treatment also was associated with cognitive impairment in patients with SCH (OR 4.01; 95% CI: 2.44–7.27; *P* < 0.0001).

Cumulative doses of antipsychotic and antidepressant agents were also associated with cognitive impairment in the entire cohort. ORs varied from 1.35 to 5.39 among the diagnostic categories (all *P* < 0.001). More interestingly, although valproate carries a lower risk of MetS, it remained an associated factor of cognitive impairment, with ORs ranging from 6.46 to 10.00 (all *P* < 0.001). In contrast, lithium demonstrated a protective effect on cognitive function; ORs varied from 0.33 to 0.66 (all *P* < 0.001). Multivariate analysis disclosed that cognitive impairment was associated with having undergone ECT among patients with SCH (OR 3.33; 95%CI 1.59–7.59; *P* < 0.0001) and BP (OR 2.88; 95% CI 2.00–5.00; *P* < 0.0001), but unrelated to the number of ECT sessions. Complete data are listed in Tables 1–4.

**Table 1:** Socio-demographic and clinical data.

	**BP** **(*N* = 787)**	**MDD** **(*N* = 899)**	**SCH** **(*N* = 1,178)**	**All** **(*N* = 2,864)**
Education (years) ≤ 12	246 (31.26)	360 (40.04)	488 (41.13)	1,094 (38.20)
>12	541 (68.74)	539 (59.96)	690 (58.87)	1,770 (61.80)
Age (years)	27.8 ± 3.9	27.0 ± 2.6	27.2 ± 4.1	27.4 ± 2.8
Illness duration (months)	42.5 ± 10.2	41.9 ± 6.9	42.4 ± 9.5	42.0 ± 7.9
Waist circumference (cm)	92.5 ± 2.8	88.5 ± 1.2	96.5 ± 4.3	94.1 ± 6.7
**Pretreatment**				
BMI	22.4 ± 2.1	21.7 ± 2.4	22.0 ± 2.9	22.2 ± 2.5
HbA1c	4.7 ± 0.3	4.4 ± 0.1	4.6 ± 0.2	4.6 ± 0.6
FBS (mmol/L)	4.6 ± 0.2	4.9 ± 0.4	4.8 ± 0.1	4.8 ± 0.4
PBG-2h (mmol/L)	6.8 ± 1.7	6.9 ± 0.9	7.0 ± 1.3	6.9 ± 0.8
HDL cholesterol (mmol/L)	1.0 ± 0.2	0.8 ± 0.1	1.2 ± 0.4	1.0 ± 0.3
Triglyceride (mmol/L)	1.3 ± 0.2	1.4 ± 0.2	1.5 ± 0.2	1.4 ± 0.4
Prolactin (ng/ml)	7.1 ± 1.7	6.9 ± 1.2	5.9 ± 2.0	6.3 ± 2.1
**4–8-week treatment interval**				
HbA1c	5.0 ± 0.2	4.3 ± 0.5	4.4 ± 0.2	4.2 ± 0.7
FBS (mmol/L)	5.6 ± 0.1	5.4 ± 0.2	5.5 ± 0.4	5.5 ± 0.6
PBG-2h (mmol/L)	6.4 ± 1.2	6.6 ± 0.9	6.5 ± 1.1	6.5 ± 1.4
HDL-cholesterol (mmol/L)	0.8 ± 0.2	0.6 ± 0.1	0.6 ± 0.1	0.7 ± 0.3
Triglyceride (mmol/L)	1.9 ± 0.2	1.7 ± 0.3	2.3 ± 0.2	2.0 ± 0.1
**8–12-week treatment interval**				
HbA1c	6.0 ± 0.2	5.9 ± 0.2	6.0 ± 0.1	6.0 ± 0.2
FBS (mmol/L)	6.2 ± 0.1	6.0 ± 0.3	6.5 ± 0.2	6.4 ± 0.2
PBG-2h (mmol/L)	7.2 ± 1.2	7.0 ± 1.9	7.5 ± 1.3	7.3 ± 1.5
HDL-cholesterol (mmol/L)	0.4 ± 0.2	0.5 ± 0.1	0.3 ± 0.0	0.4 ± 0.2
Triglyceride (mmol/L)	2.4 ± 0.1	2.7 ± 0.4	2.9 ± 0.5	2.6 ± 0.3
**Study enrollment**				
HbA1c	6.1 ± 0.1	5.8 ± 0.3	5.9 ± 0.3	6.0 ± 0.1
FBS (mmol/L)	6.7 ± 0.1	6.2 ± 0.3	6.6 ± 0.1	6.5 ± 0.2
PBG-2h (mmol/L)	7.6 ± 0.9	7.2 ± 0.8	7.8 ± 0.7	7.6 ± 1.0
HDL-cholesterol (mmol/L)	0.3 ± 0.1	0.4 ± 0.0	0.2 ± 0.0	0.3 ± 0.0
Triglyceride (mmol/L)	2.7 ± 0.2	2.6 ± 0.5	3.0 ± 0.6	2.8 ± 0.3
**Cognitive impairment**				
No	241(30.62)	410 (45.61)	690 (37.27)	1,090 (38.06)
Yes	546 (69.38)	489 (54.39)	739 (62.73)	1,774 (61.94)
	**BP**	**MDD**	**SCH**	**ANOVA**
**Pretreatment MCCB scores**				
Speed of processing	34.70 ± 2.22	35.00 ± 0.78.	35.25 ± 1.12	0.258
Attention vigilance	35.98 ± 2.13	34.66 ± 1.03	36.27 ± 0.85	0.539
Working memory	37.22 ± 1.09	38.20 ± 0.89	37.20 ± 1.36	0.398
Verbal learning	38.12 ± 1.54	37.88 ± 2.31	38.00 ± 1.69	0.400
Visual learning	34.25 ± 2.45	35.29 ± 1.28	36.13 ± 0.95	0.360
Reasoning	36.15 ± 1.10	38.26 ± 1.00	39.00 ± 1.69	0.437
Social recognition	38.99 ± 5.84	37.15 ± 3.69	32.93 ± 1.78	0.920
Composite	31.70 ± 2.00	31.89 ± 1.00	31.25 ± 0.85	0.311

**Table 2:** Treatment information, clinical outcome and post-treatment MCCB results.

**Study cohort**	**BP** **(*N* = 787)**	**MDD** **(*N* = 899)**	**SCH** **(*N* = 1,178)**	**All** **(*N* = 2,864)**
**Cumulative dosages of psychotropic medications (18 months preceding enrollment)** ^*^				
Chlorpromazine (mg)	106,904.2 ± 32,102.3	100,987.6 ± 241,152.3	365,298.4 ± 44,547.5	289,945.6 ± 101,352.4
Fluoxetine (mg)	164,00.2 ± 2,413.3	23,363.4 ± 3,200.7	12,400.6 ± 2,008.4	19,874.2 ± 1,203.5
Valproate (mg)	1,028,536.5 ± 45,682.8	200,288.9 ± 200,214.5	244,456.7 ± 14,591.6	856,987.5 ± 113,002.5
Lithium salt (mg)	512,580.4 ± 40,008.5	200,544.4 ± 41,526.5	188,940.0 ± 21,456.1	509,457.8 ± 466,414.9
Diazepam (mg)	8,018.5 ± 1,324.9	9,856.4 ± 2,045.8	4,258.6 ± 3,425.6	7,899.6 ± 3,566.2
Benzhexol (mg)	2,803.4 ± 228.9	2,936.5 ± 450.2	3,213.6 ± 994.5	3,015.2 ± 144.9
Promethazine (mg)	50,333.2 ± 14,000.5	32,533.6 ± 9,008.5	60,045.5 ± 22,004.2	58,479.1 ± 28,555.3
Aripiprazole (mg)	3,034.6 ± 484.6	2,285.64 ± 256.3	4,123.5 ± 666.6	3,526.0 ± 935.5
**Electroconvulsive therapy (18 months preceding enrollment)**				
No	616 (78.27)	601 (66.85)	638(54.16)	1,855 (64.77)
Yes	171 (21.73)	298 (33.15)	540 (45.84)	1,009 (35.23)
ECT sessions	24.5 ± 6.58	39.25 ± 16.0	45.26 ± 22.5	41.25 ± 15.00
	**BP**	**MDD**	**SCH**	***P*** **(ANOVA)**
**MCCB scores at enrollment**				
Speed of processing	25.700 ± 0.85	29.22 ± 2.00	22.99 ± 4.36	0.021
Attention vigilance	27.12 ± 0.44	27.55 ± 1.03	25.55 ± 1.25	0.0046
Working memory	29.69 ± 0.59	29.55 ± 1.25	28.25 ± 0.89	0.052
Verbal learning	29.56 ± 0.85	28.44 ± 0.78	25.40 ± 1.25	0.034
Visual learning	20.22 ± 2.23	22.258 ± 2.25	19.00 ± 1.85	0.067
Reasoning	29.78 ± 1.22	31.88 ± 1.00	28.40 ± 1.95	0.020
Social recognition	28.80 ± 1.45	31.00 ± 1.36	265.07 ± 1.66	0.010
Composite	24.18 ± 1.75	26.37 ± 1.85	23.90 ± 0.72	0.007

* Chlorpromazine and fluoxetine equivalents were used to record the cumulative dosage of antipsychotic or antidepressant agents, respectively. Sodium valproate equivalent was used to record the cumulative dosage of the mood stabilizers. Diazepam equivalent was used to calculate the cumulative dosage of the anxiolytics and sleeping agents.

**Table 3:** Univariate analysis of risk factors of cognitive impairment.

		**BP** **(*N* = 787)**	**MDD** **(*N* = 899)**	**SCH** **(*N* = 1,178)**	**All** **(*N* = 2,864)**
Variable	SCH	–	–	–	1.0
	MDD	–	–	–	1.04 (0.83–1.30)
	BP	–	–	–	1.66 (1.31–2.09)
Education (years)	≤ 12	1.0	1.0	1.0	1.0
	>12	1.18 (0.71–3.42)	1.74 (0.99–4.00)	1.22 (0.82–1.98)	1.46 (0.70–5.16)
Age (years)		2.39 (1.45–4.20)	2.15 (1.10–3.66)	2.01 (1.14–3.80)	2.30(1.10–4.57)
MPD duration at enrollment (months)		1.10 (0.89–2.30)	1.01 (0.90–1.33)	1.25 (0.98–1.92)	1.41 (0.85–2.01)
Pretreatment waist circumference		1.40 (0.97–4.56)	1.21 (0.87–3.96)	2.74 (1.33–3.47)	1.86 (0.36–5.10)
Pretreatment BMI		1.42 (0.78–2.28)	1.21 (0.93–1.99)	2.98 (1.53–3.96)	1.45 (0.76–4.25)
Pretreatment	HbA1c	9.12 (7.58–11.45)	6.11 (3.50–10.15)	8.41 (4.77–9.99)	7.10 (3.43–14.91)
	FBS	8.22(3.71–11.00)	5.77 (3.75–10.20)	7.33 (4.13–9.53)	6.99 (4.00–11.85)
	PBG-2h	3.56 (1.69–8.36)	3.86 (2.33–6.40)	9.00 (6.32–11.52)	5.59 (1.30–12.00)
	HDL cholesterol	1.50(0.43–2.55)	1.82 (0.75–3.00)	0.99 (0.54–1.94)	1.30 (0.66–4.20)
	Triglyceride	1.67 (0.84–2.30)	1.96 (0.95–3.30)	1.75 (0.88–3.02)	1.74 (0.57–3.88)
	Prolactin	1.29 (0.85–1.89)	1.20 (0.97–1.99)	1.92(1.03–3.12)	1.42 (0.79–4.03)
4–8–week treatment interval	HbA1c	7.00 (4.23–9.55)	5.27 (2.36–7.59)	7.01 (5.33–8.90)	6.27 (4.29–9.22)
	FBS	7.10(5.58–9.90)	5.48 (2.57–9.36)	6.29 (3.33–9.50)	5.99 (1.30–10.00)
	PBG−2h	7.60 (4.59–9.69)	6.80 (4.71–9.15)	8.99 (4.32–13.13)	7.59 (3.30–15.55)
	HDL cholesterol	2.50(0.97–4.88)	1.67 (0.88–5.44)	1.03 (0.77–2.00)	1.60 (0.63–6.39)
	Triglyceride	2.55 (0.99–4.22)	1.55 (0.65–3.47)	3.79 (1.88–5.44)	2.60 (0.55–6.81)
	Prolactin	1.90(0.94–2.39)	1.80 (0.77–2.19)	2.69(1.15–3.46)	2.02 (0.80–4.44)
8–12–week treatment interval	HbA1c	17.23(11.88–19.00)	11.55 (10.28–14.52)	16.17 (12.55–19.99)	15.00 (9.20–20.22)
	FBS	7.30(4.60–9.93)	7.04 (3.067–10.29)	7.00 (3.17–9.28)	7.75 (3.30–11.98)
	PBG−2h	6.35(4.14–9.33)	6.00 (4.19–9.03)	11.45 (10.40–15.28)	8.9 (4.10–16.80)
	HDL cholesterol	4.50(3.56–8.30)	2.44(1.55–5.55)	7.83 (3.99–9.69)	6.85 (1.55–9.98)
	Triglyceride	1.55 (0.90–2.00)	1.14 (0.33–3.00)	6.97 (5.66–11.11)	4.60 (0.26–16.81)
	Prolactin	2.30 (0.99–4.55)	1.97 (0.93–3.20)	4.97(3.10–7.60)	3.02 (0.80–9.44)
Chlorpromazine (cumulative dosage)^ξ^		6.85(3.48–8.69)	4.56 (2.21–9.81)	7.02 (4.54–9.81)	5.90 (1.96–10.00)
Fluoxetine (cumulative dosage)^ξ^		2.22 (0.75–3.88)	2.69 (0.96–3.89)	1.22 (0.75–2.44)	1.75 (0.75–3.99)
Valproate (cumulative dosage)^ξ^		4.99 (2.56–7.66)	8.00 (3.73–12.09)	6.87 (3.48–9.90)	6.95 (3.26–13.22)
Lithium (cumulative dosage)^ξ^		0.56 (0.28–0.76)	0.65 (0.30–0.93)	0.75 (0.55–0.97)	0.69(0.18–0.99)
Diazepam (cumulative dosage)^ξ^		1.09(0.66–2.20)	0.85 (0.66–1.24)	1.25 (1.01–1.69)	1.10 (0.37–1.96)
Benzhexol (cumulative dosage)^ξ^		1.64 (1.20–3.24)	1.72 (1.20–4.08)	2.60 (1.31–2.88)	1.99 (1.18–5.67)
Promethazine (cumulative dose)^ξ^		1.30 (0.70–2.740)	1.25 (0.69–1.95)	1.15 (0.80–1.92)	1.25 (0.97–1.92)
Aripiprazole (cumulative dose)^ξ^		2.25 (1.65–4.300)	1.06 (1.00–2.50)	1.77 (1.30–3.33)	1.98 (1.00–5.47)

ξ Using logarithmic calculation. All cumulative dosages were calculated for 18 months before enrollment.

**Table 4A:** Multivariate analysis of risk factors for cognitive impairment in all subjects.

**Risk factor**	**OR (95% CI)**	***P*** **-value**
BP vs. SCH	3.25 (2.09–5.11)	<0.0001
MDD vs. SCH	0.89 (0.44–1.21)	<0.0001
HbA1c in 8–12–week treatment interval	8.45(6.30–9.87)	<0.0001
FBS in 8–12-week treatment interval	6.29 (4.66–9.99)	<0.0001
PBG-2h in 8–12-week treatment interval	7.34 (4.55–12.12)	<0.0001
Cumulative chlorpromazine dosage	1.26 (1.05–2.85)	<0.0001
Cumulative fluoxetine dosage	5.26 (2.33–9.02)	<0.0001
Cumulative valproate dosage	3.60 (2.00–8.58)	<0.0001
Cumulative lithium dosage	0.53 (0.35–0.88)	<.00001

**Table 4B:** Multivariate analysis of risk factors for cognitive impairment in patients with BP.

**Risk factor**	**OR (95% CI)**	***P*** **-value**
HbA1c in 8–12-week treatment interval	9.95 (7.40–12.46)	<0.0001
FBS in 8–12-week treatment interval	6.52 (4.85–8.91)	<0.0001
PBG-2h in 2–3-week treatment interval	7.27 (5.68–9.89)	<0.0001
Electroconvulsive therapy	2.88 (2.00–5.00)	<0.0001
Cumulative chlorpromazine dosage	5.39 (3.21–8.88)	<0.0001
Cumulative fluoxetine dosage	1.09 (1.00–1.27)	<0.0001
Cumulative valproate dosage	10.00 (8.11–13.26)	<0.0001
Cumulative lithium dosage	0.33 (0.25–0.48)	<0.0001

**Table 4C:** Multivariate analysis of risk factors for cognitive impairment in patients with MDD.

**Risk factor**	**OR (95% CI)**	***P*** **-value**
HbA1c in 8–12-week treatment interval	8.29 (4.88–12.33)	<0.0001
FBS in 8–12-week treatment interval	4.99 (2.25–9.60)	<0.0001
PBG-2h in 8–12-week treatment interval	3.68 (1.53–7.49)	<0.0001
Cumulative chlorpromazine dosage	3.00 (1.85–5.70)	<0.0001
Cumulative fluoxetine dosage	1.44 (1.00–2.13)	<0.0001
Cumulative valproate dosage	6.46 (4.42–9.25)	<0.0001
Cumulative lithium dosage	0.42 (0.22–0.63)	<0.0001

**Table 4D:** Multivariate analysis of risk factors for cognitive impairment in patients with SCH.

**Risk factor**	**OR (95% CI)**	***P*** **-value**
Waist circumference pretreatment	4.01 (2.44–7.27)	<0.0001
HbA1c in 8–12-week treatment interval	6.66 (4.29–8.49)	<0.0001
FBS in 8–12-week treatment interval	6.59 (4.28–9.99)	<0.0001
PBG-2h in 8–12-week treatment interval	8.20 (6.40–10.00)	<0.0001
Triglyceride in 4–12-week treatment interval	6.96 (4.19–9.97)	<0.0001
HDL-cholesterol in 8–12-week treatment interval	5.38 (2.54–8.25)	<0.0001
Electroconvulsive therapy	3.33 (1.59–7.59)	<0.0001
Cumulative chlorpromazine dosage	5.28 (2.60–9.65)	<0.0001
Cumulative fluoxetine dosage	1.35 (1.00–2.67)	<0.0001
Cumulative valproate dosage	9.75 (6.86–11.99)	<0.0001
Cumulative Lithium dosage	0.66 (0.45–0.97)	<0.0001

## Discussion

Four valuable findings of this retrospective study may inform treatment strategies to reduce the suffering of young women with MPD. The first is that cognitive impairment was highly prevalent (68.75%) in our study cohort, with rates of 69.38, 62.73, and 54.39% among young women with BP, SCH, and MDD, respectively. Over 50% of patients developed cognitive impairment within the first 1.5 years of MPD onset. Sub-group analysis revealed that visual learning ability declined more acutely than the other six dimensions of MCCB in patients with BP, SCH, and MDD. These convergent lines of evidence suggest a high prevalence of cognitive impairment in patients with MPD, and that the visual learning dimension is affected most severely. The score reduction of over 50% indicates that half of visual learning ability was lost. In addition, the scores of the entire cohort in all seven dimensions of the MCCB were 1.5–2 standard deviations lower than the Chinese norm [Global deficit Scores (GDS) >3, when compared to the Chinese norm]. These findings suggest that cognitive impairment precedes MPD onset, supporting the neurodevelopment hypothesis of MPD.

The second major finding is that the highest cognitive impairment was observed in patients with BP, and was associated with highly significant elevations of HbA1C, FBS, and FGB-2h within the first 2–3 months of psychotropic therapy.

The third major finding of our study is that cumulative dosages of chlorpromazine, fluoxetine and valproate equivalents were risk factors of cognitive impairment. The OR of valproate was highest, whether used as an anti-mania therapy or as a synergistic agent to improve depressive or psychotic symptoms, suggesting that the serious side-effect of cognitive impairment should be addressed in clinical practice. Previous studies have reported that valproate decreased cognitive performance in patients with BP, SCH, and MDD (43, 69). Although some studies suggested that the addition of valproate to antipsychotics may improve cognitive function in patients with SCH, recent evidence has been generally weak. For example, a randomized controlled trial of adjunctive valproate for cognitive remediation in early SCH demonstrated that the effect of valproate was equivalent to placebo (70, 71). More importantly, when used as a synergistic drug to improve depressive symptoms, valproate was also linked to impaired cognition. These convergent lines of evidence indicate that the risk-benefit ratio of valproate should be carefully considered when selecting patient candidates for valproate treatment. The mechanism of valproate-induced cognitive dysfunction may be related to a drug-induced disturbance of glycogen synthase kinase3β (GSK-3β) phosphorylation (72, 73), although further studies are needed for clarification.

The fourth important finding of our study is that lithium preserved cognition in all MPD subgroups, both as a monotherapy and when used as a synergistic agent. Multiple studies have reported that lithium exerts neuroprotection. For example, Ochoa et al. reported that lithium is an effective neuroprotectant for patients with BP, especially for improving cognition by modulating nerve growth factors, inflammation, mitochondrial function, oxidative stress, and programmed cell death mechanisms such as autophagy and apoptosis (74). Additionally, Puglisi-Allegra et al. reported that adjunctive lithium alleviated the cognitive impairments of other psychiatric disorders (75). Collectively, the findings of previous reports and our study converge to indicate that lithium can preserve cognitive ability; hence, lithium should be recommended as an adjunct to other psychotropic agents to mitigate cognitive impairment.

The fifth important finding of our study is that decreased GDS scores were related to ECT administration, but unrelated to the number of ECT sessions, suggesting that ECT is a risk factor for cognitive impairment.

## Limitations

The first and most important limitation of our study is that recall bias could not be fully eliminated, even though our participants were screened for normal memory function by Wechsler Memory Scale testing. Although this remedial measure may not permit the same strength of evidence that might result from a prospective study, our findings may inform clinical practice. The second limitation is that although our data demonstrated that elevated indices of blood glucose levels indicate increased risk of cognitive impairment, HbA1c reflects glycemic control only during the preceding 3 rather than 18 months. Whether HbA1c can serve as a biomarker for the risk of cognitive impairment needs further prospective studies for confirmation. However, in patients with MPD, the relationship between hyperglycemia and therapeutic agents has been confirmed by multiple studies; hence, our data are inclined to support an association between elevated HbA1c levels and deterioration of cognitive function, although further studies of pathogenesis are needed for clarification. The third limitation is that the prevalence of cognitive impairment was obviously higher in patients with BP than among those with SCH and MDD. Although valproate effects on GSK-3β may explain cognitive impairment, further neurotoxicological studies are needed to confirm an underlying mechanism and to close the argument that valproate exerts varying cognitive effects in different studies. The fourth limitation is that our data could not demonstrate that cognitive impairment before the onset of MPD was unrelated to the cognitive status during the 18 months study period; hence, a prospective study is needed for clarification. The fifth limitation is that nearly one fourth of our participants accepted ECT treatment. Our data demonstrated that cognitive impairment was related to whether or not ECT was administered, but unrelated to the number of ECT sessions. The sixth limitation is that our data demonstrated that hyperprolactinemia was unrelated to cognitive impairment. A limited number of studies have reported a relationship between hyperprolactinemia and cognition; hence, this phenomenon requires further study. The seventh limitation is that due to its retrospective design, our findings cannot describe the interaction relationship of the “hyperglycemia-cognitive impairment” cycle adequately. The eighth limitation is that our data cannot explain the differences of cognitive impairment associated with multiple factors among women with SCH, BP, and MDD. The ninth limitation is that enrollment was limited to female patients. This enrollment strategy was undertaken because male patients with major psychiatric disorder usually have long-term histories of alcohol abuse or nicotine dependence. Previous studies have confirmed that chronic alcohol and nicotine use alter cerebrovascular function and impair glycemic control, and consequently degrade cognitive ability. Thus, a high prevalence of alcohol and nicotine use among male subjects would have introduced confounding variables into our study. Although we did not evaluate male patients, we hypothesize that they may experience cognitive impairments related to alcohol and nicotine use.

## Conclusion

Our study offers five insights that may inform clinical practice. The first is that elevated HbA1c within the first 8–12-week interval of psychotropic therapy in young women with MPD was associated with poor cognitive performance. The second is that hypertriglyceridemia within the 8–12-week treatment interval was also associated with cognitive decline. The cumulative dosages of therapeutic agents were also associated with cognitive impairments. Waist circumference in the 8–12-week treatment interval also was a risk factor of cognitive impairment in SCH. The monitoring of these indexes may guide treatment revisions to improve clinical outcomes. The third is that patients with BP exhibited the highest risk of cognitive impairment due to drug-induced hyperglycemia. The fourth is that valproate remained a risk factor of cognitive impairment. Our findings suggest that clinicians should monitor their patients for the development of hyperglycemia within the first 8–12 weeks of psychotropic treatment.

## Data availability statement

The original contributions presented in the study are included in the article/supplementary material, further inquiries can be directed to the corresponding author/s.

## Ethics statement

The studies involving human participants were reviewed and approved by Tianjin Fourth Center Hospital Committee of IRB. The patients/participants provided their written informed consent to participate in this study.

## Author contributions

WL, XS, RJ, and CZ conceived and designed research. RL, HY, GC, JS, JZ, ZC, CLin, LC, YX, SL, QLi, SJ, CLiu, QZ, LY, JC, QLuo, LW, HT, and CZ collected data and conducted research. JC, QLuo, LW, HT, and CZ analyzed and interpreted data. LW, HT, and CZ wrote the initial paper. QLuo, SJ, CLin, and CZ revised the paper. CZ and HT had primary responsibility for final content. All authors read and approved the final manuscript.

## Funding

This work was supported by grants from the National Natural Science Foundation of China (81871052, 82171503 to CZ), the Key Projects of the Natural Science Foundation of Tianjin, China (17JCZDJC35700 to CZ), the Tianjin Health Bureau Foundation (2014KR02 to CZ), and the Tianjin Science and Technology Bureau (15JCYBJC50800 to HT).

## Conflict of interest

The authors declare that the research was conducted in the absence of any commercial or financial relationships that could be construed as a potential conflict of interest.

## Publisher's note

All claims expressed in this article are solely those of the authors and do not necessarily represent those of their affiliated organizations, or those of the publisher, the editors and the reviewers. Any product that may be evaluated in this article, or claim that may be made by its manufacturer, is not guaranteed or endorsed by the publisher.

## Abbreviations

HbA1c: Glycosylated hemoglobin
MPD: Major psychiatric disorder
MetS: Metabolism syndrome
SCH: Schizophrenia
BP: Bipolar disorder
MDD: Major depressive disorder
MCCB: MATRICS Consensus Cognitive Battery
ORs: Odds ratios
DSM-IV-TR: Diagnostic and Statistical Manual of Mental Disorders, Fourth Edition, Text Revision
BIS: Birchwood Insight Scale
BCIS: Beck Cognitive Insight Scale
SCID-I/P: Statistical Manual of Mental Disorders Fourth Edition, Text Revision Axis I Disorders, Research Version, Patient Edition
BMI: Body Mass Index
FBS: Fasting blood sugar
PBG-2h: 2-hour postprandial blood glucose
CI: Confidence intervals
GSK-3β: Glycogen synthase kinase3β
ECT: Electroconvulsive therapy.
